# Complete chloroplast genome of *Reineckia carnea* and its implications for the phylogenetic position within Nolinoideae (Asparagaceae)

**DOI:** 10.1080/23802359.2019.1623119

**Published:** 2019-07-10

**Authors:** Zhong-Shuai Sun, Peng-He Cao, Yue-Ling Li, Sheng Huang

**Affiliations:** a Zhejiang Provincial Key Laboratory of Plant Evolutionary Ecology and Conservation, Taizhou University, Taizhou, China;; b Department of Bioengineering, Enshi Polytechnic, Enshi, China;; c College of Forestry and Horticulture, Hubei Minzu University, Enshi, China

**Keywords:** *Reineckia carnea*;·Nolinoideae, chloroplast genome, phylogenomics

## Abstract

*Reineckia carnea* is an important horticultural and medicinal plant in East Asia. Here, we determined the first complete chloroplast genome of *R. carnea* using genome skimming approach. The cp genome was 157,059 bp long, with a large single-copy region (LSC) of 85,474 bp and a small single-copy region (SSC) of 18,535 bp separated by a pair of inverted repeats (IRs) of 26,525 bp. It encodes 132 genes, including 86 protein-coding genes, 38 tRNA genes, and eight ribosomal RNA genes. The phylogenetic analysis indicated that *R. carnea* is close related to *Rohdea chinensis*.

*Reineckia carnea* (Andr.) Kunth is the sole species of its genus in Asparagaceae (Tsukamoto 1988). It mainly distributed in China and Japan of East Asia and is an important horticultural plant and also a folk medicine in southwestern China for centuries (Zhang 2009; Han et al. 2010). The classification of the Nolinoideae (Asparagaceae) has long been problematic, generic-level phylogenetic studies using a limited set of markers have often not been able to fully resolve relationships within this plant family, indicating that a higher number of molecular characters are required for an improved understanding of relationships within this group (Seberg et al. 2012; Meng et al. 2014; Wang et al. 2014). Therefore, we sequenced the whole chloroplast genome of *R. carnea* to elucidate its phylogenetic relationship within Nolinoideae (Asparagaceae).

Total genomic DNA was extracted from silica-dried leaves collected from Maoba-town, Lichuan city in western of Hubei province, China using a modified CTAB method (Doyle and Doyle 1987). The voucher specimen (SZ010) was collected and deposited in the Herbarium of Taizhou University. DNA libraries preparation and pair-end 125 bp read length sequencing were performed on the Illumina HiSeq 2500 platform. About 7.8 Gb of raw data were trimmed and assembled into contigs using CLC Genomics Workbench 8. Then, all the contigs were mapped to the reference cp genome of *Rohdea chinensis* (MH356725; Zhou et al. 2018) using BLAST (NCBI BLAST v2.2.31) search and the draft *cp* genome of *Reineckia carnea* was constructed by connecting overlapping terminal sequences in Geneious R11 software (Biomatters Ltd., Auckland, New Zealand). Gene annotation was performed via the online program Dual Organellar Genome Annotator (DOGMA; Wyman et al. 2004).

The complete cp genome of *R. carnea* (GenBank accession MK801116) was 157,059 bp long consisting of a pair of inverted repeat regions (IRs with 26,525 bp) divided by two single-copy regions (LSC with 85,474 bp; SSC with 18,535 bp). The overall GC content of the total length, LSC, SSC, and IR regions were 37.6%, 35.6%, 31.5%, and 43.0%, respectively. The genome contained a total of 132 genes, including 86 protein-coding genes, 38 tRNA genes, and eight rRNA genes.

To determine the phylogenetic position of newly sequenced *R. carnea*, phylogenetic analysis was conducted along with 11 representative *Nolinoideae* species and two outgroup taxa. We reconstructed a phylogeny employing the GTR + G model and 1000 bootstrap replicates under the maximum-likelihood (ML) inference in RAxML-HPC v.8.2.10 on the CIPRES cluster (Miller et al. 2010). The ML tree (Figure 1) was consistent with the most recent phylogenetic study on Polygonateae (Asparagaceae) (Floden and Schilling 2018). *Reineckia carnea* exhibited the closest relationship with *Rohdea chinensis*.

**Figure 1.**
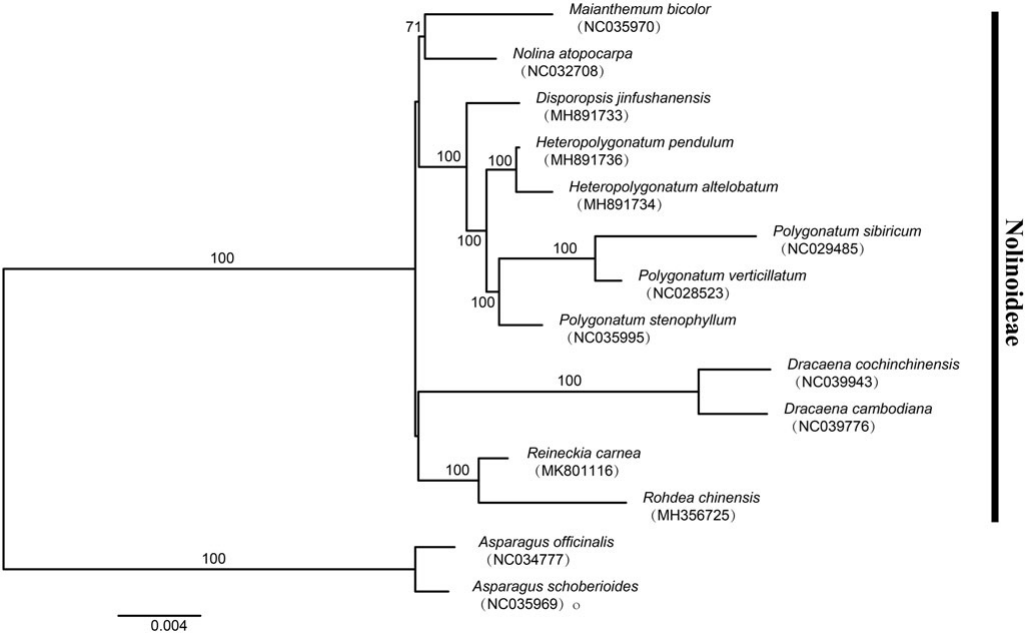
Phylogenetic tree reconstruction of 11 taxa of *Nolinoideae* (Asparagaceae) and two outgroups using ML method. Relative branch lengths are indicated. Numbers near the nodes represent ML bootstrap value.
